# The sensitivity and specificity of the modified volume-viscosity swallow test for dysphagia screening among neurological patients

**DOI:** 10.3389/fneur.2022.961893

**Published:** 2022-09-16

**Authors:** Yiqiu Lin, Guifang Wan, Huixiang Wu, Jing Shi, Yaowen Zhang, Huayu Chen, Xiaomei Wei, Zhiming Tang, Meng Dai, Zulin Dou, Hongmei Wen

**Affiliations:** Department of Rehabilitation Medicine, The Third Affiliated Hospital, Sun Yat-sen University, Guangzhou, China

**Keywords:** dysphagia, modified volume-viscosity swallow test, screening, neurological diseases, sensitivity, specificity

## Abstract

Oropharyngeal dysphagia (OD) is a highly prevalent condition after stroke and other neurological diseases. The volume-viscosity swallow test (V-VST) is a screening tool for OD. Considering that the recommendations of volume and thickeners in the original V-VST limited the popularization and application of the test in the Chinese population, we provide the modified V-VST to detect OD among neurological patients. In addition, the accuracy of the modified V-VST to screen OD needs to be verified. We included 101 patients with neurological diseases. OD was evaluated by a modified V-VST and a videofluoroscopy swallowing study (VFSS) using 3 volumes (i.e., 3, 5, and 10 ml) and 4 viscosities (i.e., water, mildly thick, moderately thick, and extremely thick). In this study, to compare with the original V-VST results, a volume of 20 ml was also included. The discriminating ability of modified V-VST in detecting OD was assessed by the sensitivity and specificity values of clinical signs of impaired efficiency (impaired labial seal, piecemeal deglutition, and residue) and impaired safety of swallowing (cough, voice changes, and oxygen desaturation ≥3%) in comparison to the results of VFSS. The modified V-VST showed 96.6% sensitivity and 83.3% specificity for OD, 85.2% sensitivity and 70% specificity for impaired safety, and 90.9% sensitivity and 76.9% specificity for impaired efficacy. Our study suggests that the modified V-VST offers a high discriminating ability in detecting OD among neurological patients.

## Introduction

Oropharyngeal dysphagia (OD) is a highly prevalent condition after stroke and other neurological diseases (1–4) and can cause serious complications including malnutrition (5), dehydration, aspiration pneumonia (6), and premature mortality (1, 7, 8). A decrease in deglutition may increase the risk of aspiration, pneumonia, and death (9). Despite its high prevalence among vulnerable patients and associated serious complications, dysphagia is often overlooked and underdiagnosed in vulnerable patient populations (1, 10).

Clinical screening methods should be used to identify OD and to identify those patients who are at risk of aspiration (11). Videofluoroscopy swallowing study (VFSS) is the gold standard to explore the oral and pharyngeal mechanisms underlying swallowing dysfunction (12). With its visual characteristics, VFSS helps clinical medical staff find problems when patients swallow. Considering the dependence of VFSS on specific equipment, easy and quick clinical screening or assessment methods are necessary. Obviously, questionnaires are widely used screening methods in clinical practice (13). One such screening tool is the Eating Assessment Tool (EAT-10), a questionnaire used to detect dysphagia symptoms in a population with a wide range of causes of swallowing difficulties (14). Meanwhile, the water swallow test is also an easy test to detect swallowing difficulties (15). However, the limited specificity and sensitivity of these tests and the potential unsafety risk of large amounts of water swallowing further prevent them from widespread use (16, 17).

Previous studies (17–20) have provided evidence about the sensitivity and specificity of the volume-viscosity swallow test (V-VST) for detecting dysphagia. For impaired safety of swallowing, the V-VST presented a sensitivity of 84.2–88.2% and a specificity of 64.7–81.0%. Specifically, the V-VST showed 88.2–100% sensitivity and 28–71.4% specificity for aspiration and 34.3–83.7% sensitivity and 64.7–70.6% specificity for penetration (17–19). For impaired efficacy, the V-VST presented 79% sensitivity and 75% specificity (19). These results suggest that the V-VST seems to be a valuable screening tool for detecting OD.

The V-VST uses swallow boluses of various volumes (5, 10, and 20 ml) and viscosities (thin liquid, nectar-like, and spoon thick) and was first validated against VFSS by using liquids thickened with a starch-based thickener (18). However, in previous clinical practice, we found that even with the smallest volume of 5 ml, many severe swallowing difficulty patients showed aspiration or penetration. Given that 3 ml is a common parameter in swallowing screening and assessment tests (21–24), we added a 3 ml volume to the test as initial volume. Moreover, starch-based thickeners present some limitations in taste (25), and hydrolysis caused by contact with amylase in the saliva leads to the change of viscosities and impaired accuracy of the test (26–28). Therefore, it is necessary to modify the V-VST in the bolus volume tested and the thickener used.

We modified the V-VST as follows: The modified volume-viscosity swallow test (modified V-VST) starts with 3 ml instead of 5 ml and ends with 10 ml. Instead of starch-based thickeners, we used xanthan gum-based thickeners, and four viscosities (i.e., water, mildly thick, moderately thick, and extremely thick) were used according to the Classification of Modified Diet for Dysphagic Persons in 2013 of the Japanese Society of Dysphagia Rehabilitation (JSDR) (29, 30). The aim of this study was to validate the accuracy of the clinical bedside screening test modified V-VST in the detection of OD.

## Materials and methods

### Patients

The patients were consecutively recruited from the Department of Rehabilitation Medicine, the Third Affiliated Hospital of Sun Yat-sen University between 1 October 2018 and 31 December 2019. Patients were eligible for the study if they were diagnosed with neurological disease confirmed by computerized tomography (CT) or magnetic resonance imaging (MRI), aged >18 years. We excluded patients who had tracheal intubation, a Glasgow Coma Scale score of <10 (excluded also if motor function <6 or eye-opening <3). The study protocol was approved by the Ethics Committee of the Third Affiliated Hospital, Sun Yat-sen University. All patients gave their informed consent to all the study procedures.

### Procedure

The OD was evaluated in all patients by modified V-VST and VFSS. The trained speech-language pathologists administered the VFSS and modified V-VST to all patients. They had received professional training regarding the knowledge and practice procedure of the VFSS and modified V-VST. The modified V-VST was performed first, and a VFSS study was followed within 24 h by speech-language pathologists blinded to the results of the modified V-VST.

### The modified V-VST

Generally, four viscosities (i.e., water, mildly thick, moderately thick, and extremely thick) and three volumes (i.e., 3, 5, and 10 ml) were used in the modified V-VST.

There were two changes from the original version.

(1) Patients with neurological disease present increased oral transit duration; starch-based thickeners can hydrolyze upon contact with amylase in the saliva and break down during the oral preparatory and oral phases of deglutition (26, 27), which may lead to the change in viscosities and impaired accuracy of the test. Thus, the modified V-VST used xanthan gum thickener, which cannot be hydrolyzed by saliva and have a better stable viscosity (31, 32).

(2) The modified V-VST started with 3 ml and ended with 10 ml. In previous clinical practice, we found that even with the smallest volume of 5 ml, many patients showed aspiration. A previous study (33) also showed that it was hard to swallow a 20 ml bolus for patients with OD, and therefore, we removed the max volume of 20 ml.

In this study, to compare with the original V-VST results, a volume of 20 ml was also included.

### Bolus preparation

Four viscosities (water, IDDSI: Level 0; mildly thick, IDDSI: Level 1; moderately thick, IDDSI: Level 2; and extremely thick, IDDSI: Level 3) were used during modified V-VST and VFSS according to the viscosity ranges of the Japanese Society of Dysphagia Rehabilitation 2013 (29, 30). IDDSI flow test was performed as previously described (34) by testing liquid flows through a 10 ml slip tip syringe leaving volume in the syringe after 10 s.

For modified V-VST studies, all viscosity series were made up of Softia S thickener (Nutri Co., Ltd., Japan) and water (mildly thick: 3 g thickener/300 ml water; moderately thick: 3 g thickener/150 ml water; extremely thick: 3 g thickener/100 ml water). For VFSS studies, boluses were made by Softia S thickener and 60% w/v barium sulfate suspension (200 g of barium sulfate dissolved in 289 ml of water). The proportion of thickener and suspension was the same as that of modified V-VST. The solutions were prepared at room temperature 5 min before modified V-VST and 3 h before the videofluoroscopic examination to obtain stable and equivalent viscosities (35).

### Procedure of screening

**Figure 1:**
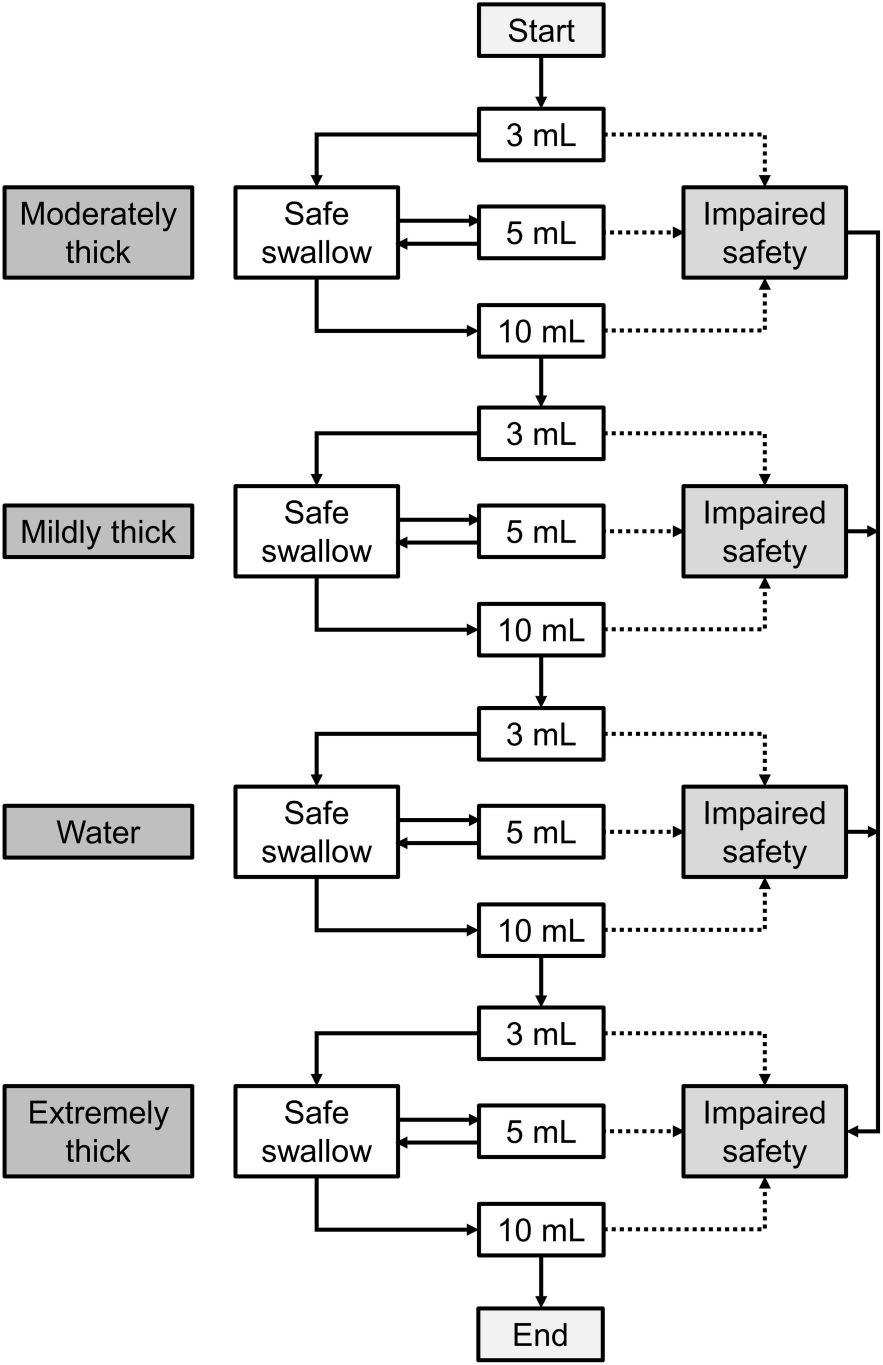
The procedure of modified V-VST. The test starts with moderately thick and increasing volumes from 3 to 5 ml and 10 ml boluses in a progression of increasing difficulty. When patients completed all bolus without signs of impaired safety (cough, changes in voice, or fall in oxygen saturation ≥3%), a test was conducted in the order of moderately thick, mildly thick, water, and extremely thick series (3 to 10 ml). If the patient presented any sign of impaired safety at the moderately thick level, the series was interrupted, the mildly thick and water series were omitted, and an extremely thick series was assessed. Similarly, if the patient presented signs of impaired safety at mildly thick, then the water series was interrupted and the extremely thick series was assessed.

Referring to the original V-VST procedure (18), the modified V-VST test starts with moderately thick and increasing volumes from 3 to 5 ml and 10 ml boluses in a progression of increasing difficulty. To compare with the original V-VST results, a volume of 20 ml was also tested (Figure 1).

The clinical signs and symptoms of OD were evaluated as described previously (36). (1) The signs of impaired efficiency of swallowing were impaired labial seal, piecemeal swallow, oral residue, and symptoms of pharyngeal residue. (2) The signs of impaired safety of swallowing were changes in voice quality, cough, and a decrease in oxygen saturation ≥3% from the basal level (measured with a finger pulse-oximeter).

A patient who presented one or more signs of impaired efficiency and/or safety of swallowing was considered to have OD. Safety of swallowing was expressed as the percentage of patients who could swallow without clinical signs of cough, changes in voice, or a fall in oxygen saturation ≥3%.

### Videofluoroscopic swallowing study (VFSS)

All patients were imaged for the videofluoroscopic study while seated in a lateral projection that included the oral cavity, pharynx, larynx, and cervical esophagus. VFSS was conducted using a gastrointestinal X-ray machine (Toshiba DBA-300, Toshiba Co. Ltd., Tokyo, Japan). Videos were recorded using a VFSS digital acquisition unit (Longest Ltd., Inc., Guangzhou, China) at 30 frames/s. The ability of the patient to swallow boluses of various volumes and viscosities was evaluated following the same strategy as that in the modified V-VST.

(1) An impairment of the safety of swallowing was considered when penetration or aspiration was detected. (2) An impairment of the efficiency of swallowing was considered when at least one of the following signs was identified during the videofluoroscopic study: impaired labial seal closure, oral residue, pharyngeal residue, or piecemeal deglutition. A patient who presented impairment in the efficiency and/or the safety of swallowing was considered to have OD.

### Data analysis and statistical methods

Quantitative parameters were described as the mean ± standard deviation (SD), and qualitative parameters were described by frequencies. The prevalence of clinical and VFSS signs was reported as the ratio between the number of each clinical or VFSS sign divided by the total number of swallows for each bolus type (any particular volume/viscosity). The prevalence of safe swallows was described as the number of patients without any sign of impaired safety of swallowing divided by the total number of patients who swallowed the bolus. The effect of bolus volume or viscosity on the safety and efficiency of swallowing was assessed by the non-parametric Cochran Q test. *P* < 0.05 was considered to be statistically significant. When the results were significantly different, for multiple statistical comparisons, we performed McNemar's chi-squared test by applying the Bonferroni corrected alpha level (corrected α = 0.05/6 compared pairs = 0.0083). To assess the accuracy of the modified V-VST relative to the VFSS, sensitivity, specificity, positive and negative predictive values, as well as positive and negative likelihood ratios were measured. 95% confidence intervals (CIs) were expressed.

## Results

### Patients

**Figure 2:**
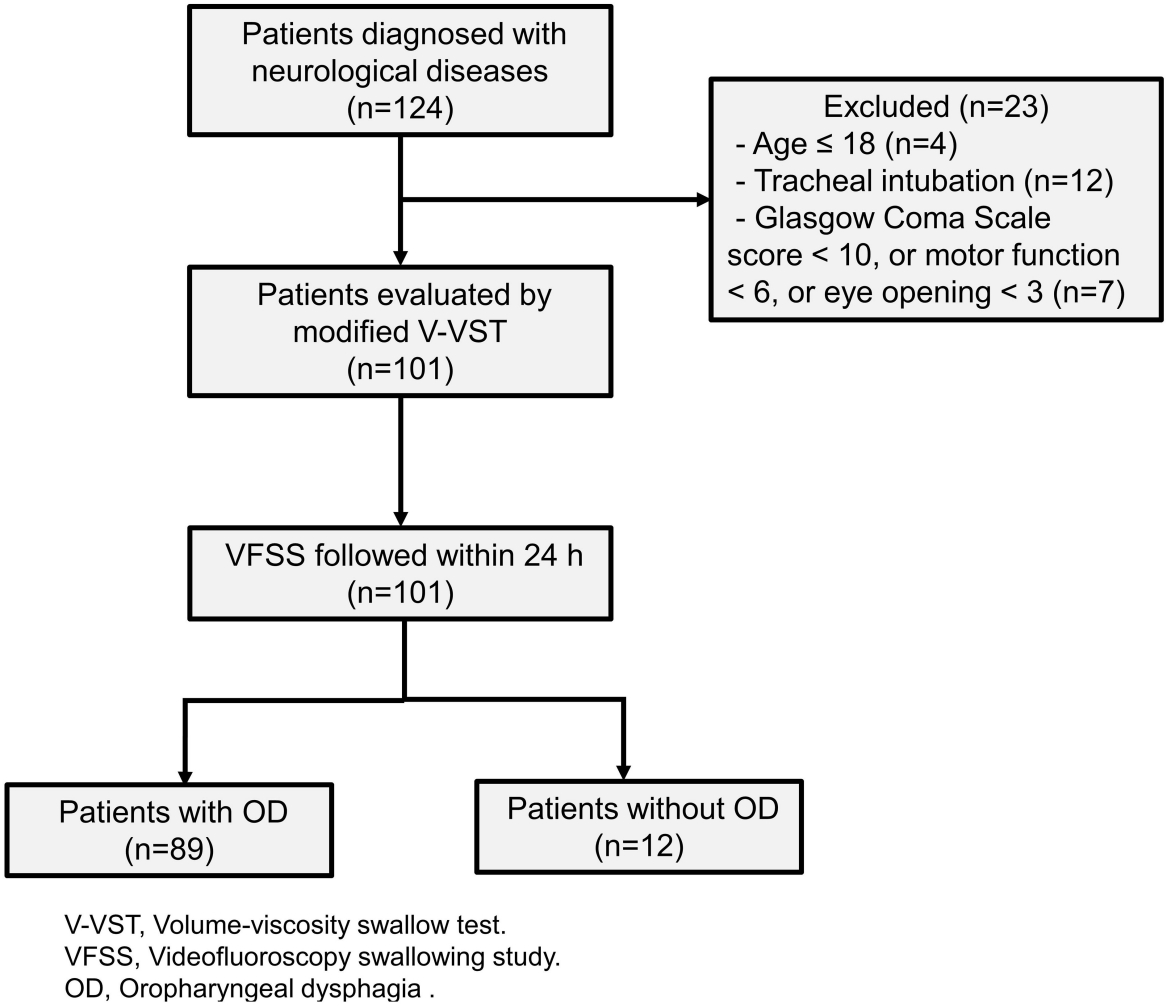
Flow diagram of included patients with neurological diseases.

Our study included 101 patients with neurological diseases (Figure 2). The etiology of these patients includes stroke, brain tumors, head and neck tumors, brain injury, and other neurological diseases including traumatic brain injury, encephalitis, and encephalomyelitis. We used the Functional Oral Intake Scale (FOIS) (37) to assess the severity of the patient's dysphagia. The results were as follows: Level 1, 15 (14.9%); Level 2, 27 (26.7%); Level 3, 10 (9.9%); Level 4, 6 (5.9%); Level 5, 18 (17.8%); Level 6, 9 (8.9%); and Level 7, 16 (15.8%). Demographic and clinical data were shown in Table 1.

**Table 1:** Demographical and clinical data of the study population.

**Subjects**	***N***	**Age (years)**	**Sex (% men)**
Patients without Dysphagia	12	52.4 ± 12.1	50% (6)
Patients with Dysphagia	89	55.1 ± 16.6	77.5% (69)
Ischemic Stroke	38	61.9 ± 13.0	81.5% (31)
Hemorrhagic Stroke	16	57.3 ± 12.9	93.7% (15)
Brain tumor	16	41.3 ± 15.4	68.7% (11)
Other neurological diseases	19	51.3 ± 19.6	63.1% (12)

### Videofluoroscopic study

According to the VFSS, the prevalence of OD in the patients was 88.1% (89); 87.1% (88) showed impaired efficiency of swallowing, and 80.2% (81) showed impaired safety of swallowing.

#### Efficiency signs

Pharyngeal residue and piecemeal swallowing were common VFSS signs in patients with dysphagia. Impaired labial seal closure, piecemeal swallowing, oral residue, and pharyngeal residue were observed in 24.8% (25), 78.2% (79), 36.6% (37), and 82.2% (83) of the patients, respectively. The prevalence of pharyngeal residue for a given bolus volume varied among the moderately thick and extremely thick bolus viscosities (*P* < 0.05). Extremely thick boluses resulted in an increased prevalence of pharyngeal residue compared with water boluses for the 5 ml and 10 ml swallows (*P* < 0.05). Increasing bolus volume increased the occurrence of piecemeal swallowing, with rates of 43.4–59.8%, 44.2–68.2%, 53.3–78.7%, and 70–84.5% for the 3 ml, 5 ml, 10 ml, and 20 ml swallows, respectively (*P* < 0.05), and these rates were unaffected by bolus viscosity.

#### Safety signs

**Figure 3:**
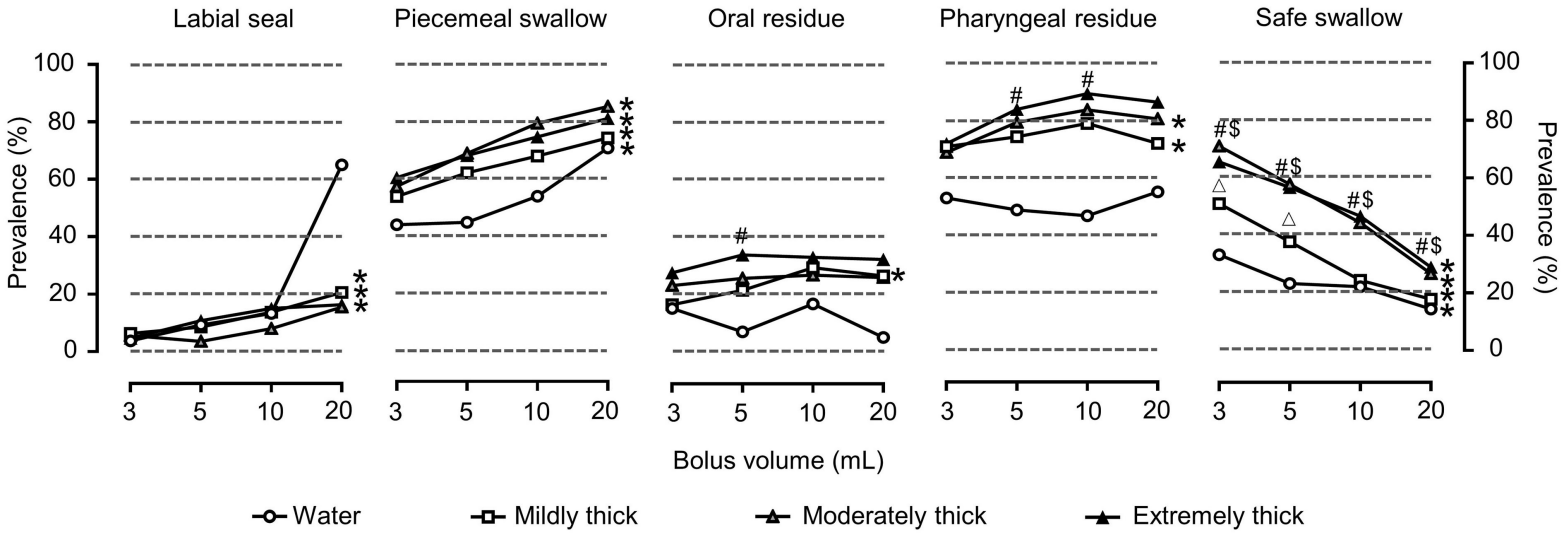
Prevalence of OD with VFSS signs of impaired efficiency and safe swallowing for each volume, viscosity, and thickener. *P < 0.05 effect of bolus volume; #*P* < 0.0083 extremely thick vs. water; $*P* < 0.0083 moderately thick vs. water; Δ*P* < 0.0083 mildly thick vs. water.

Of the patients included in the study, aspiration was the most prevalent cause of unsafe deglutition and was observed in up to 65.4% (66) of the patients, 33.3% (22) of which were silent, and penetration occurred in 14.9% (15) of the patients. Increasing bolus volume significantly impaired the safety of swallowing at all viscosities (*P* < 0.05), and the prevalence of safe swallowing significantly increased with viscosity (*P* < 0.05) (Figure 3).

### The modified V-VST

Of the patients, 87.1% (88) presented OD according to the modified V-VST, and 82.2% (83) and 74.3% (75) showed impaired efficiency and safety of swallowing, respectively.

#### Efficiency signs

**Figure 4:**
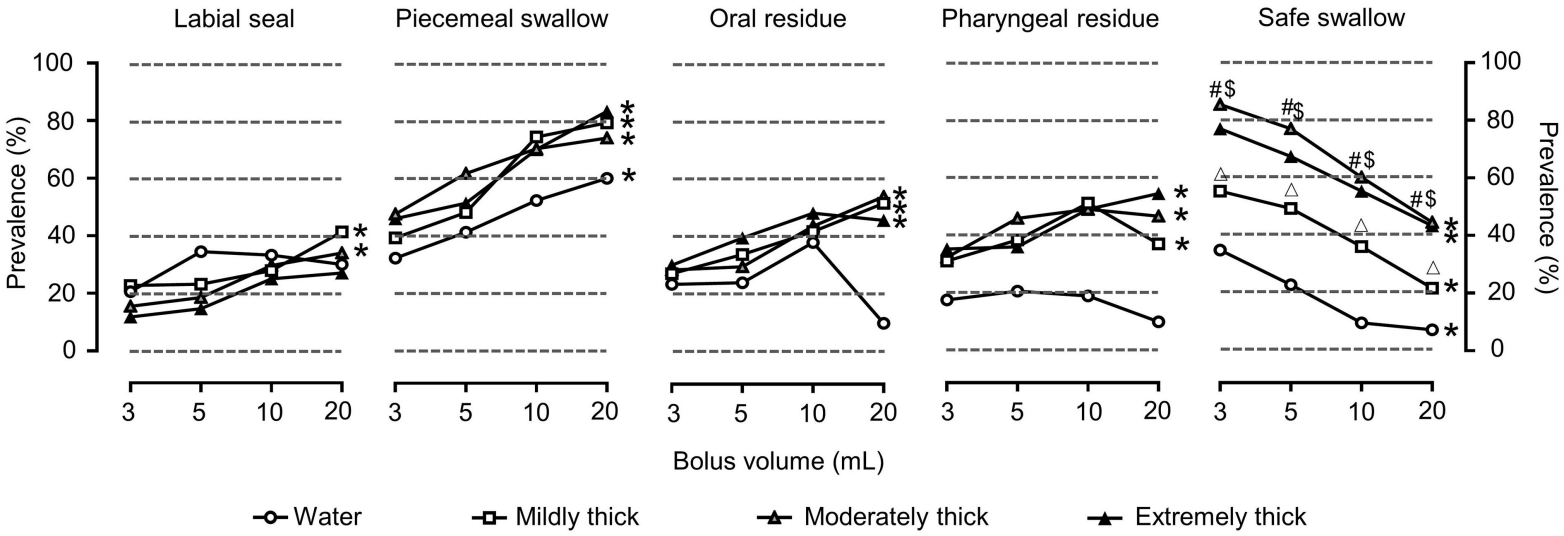
Prevalence of OD with clinical signs (modified V-VST) of impaired efficiency and safe swallowing for each volume, viscosity, and thickener. *P < 0.05 effect of bolus volume; #*P* < 0.0083 extremely thick vs. water; $*P* < 0.0083 moderately thick vs. water; Δ*P* < 0.0083 mildly thick vs. water.

The main clinical signs of impaired efficiency in OD were piecemeal swallowing and pharyngeal residue (Figure 4).

#### Safety signs

Cough was the most prevalent clinical sign and was observed in 55.4% (56) of the patients, followed by voice change in 51.5% (52) and oxygen desaturation in 36.6% (37).

### Accuracy of the modified V-VST for detecting OD

The accuracy of the modified V-VST in terms of sensitivity, specificity, predictive values, and likelihood ratios is presented in Table 2. The modified V-VST showed 96.6% sensitivity and 83.3% specificity for OD, 90.9% sensitivity and 76.9% specificity for impaired efficiency, and 85.2% sensitivity and 70% specificity for impaired safety of swallowing (aspiration or penetration). Specifically, the modified V-VST showed a sensitivity of 89.4% and a specificity of 70% for aspirations and a sensitivity of 66.7% and a specificity of 70% for penetration. For the clinical signs of impaired safety of swallowing in modified V-VST, the sensitivity and specificity of cough, voice change and oxygen desaturation by ≥3% were 65.4 and 85%, 58.0 and 75%, and 43.2 and 90%, respectively.

**Table 2:** Accuracy of the modified V-VST to detect OD.

	***Se* (95%*CI*)**	***Sp* (95% *CI*)**	***PPV* (95% *CI*)**	***NPV* (95% *CI*)**	***LHR*+ (95% *CI*)**	***LHR*– (95% *CI*)**
OD	96.6 (90.5–99.3)	83.3 (51.6–97.9)	97.7 (92–99.7)	76.9 (46.2–95)	5.8 (1.64–20.56)	0.04 (0.01–0.13)
Impaired safety	85.2 (75.6–92.1)	70 (45.7–88.1)	92 (83.4–97)	53.9 (33.4–73.4)	2.84 (1.44–5.58)	0.21 (0.12–0.38)
Aspiration	89.4 (79.4–95.6)	70 (45.7–88.1)	90.8 (81–96.5)	66.7 (43–85.4)	2.98 (1.52–5.85)	0.15 (0.07–0.32)
Penetration	66.7 (38.4–88.2)	70 (45.7–88.1)	62.5 (35.4–84.8)	73.7 (48.8–90.9)	2.22 (1.04–4.75)	0.48 (0.22–1.03)
Cough	65.4 (54–75.7)	85 (62.1–96.8)	94.6 (85.1–98.9)	37.8 (23.8–53.5)	4.36 (1.52–12.53)	0.41 (0.29–0.58)
Voice change	58 (46.5–68.9)	75 (50.9–91.3)	90.4 (79–96.8)	30.6 (18.3–45.4)	2.32 (1.06–5.07)	0.56 (0.39–0.8)
Desaturation by ≥3%	43.2 (32.2–54.7)	90 (68.3–98.8)	94.6 (81.8–99.3)	28.1 (17.6–40.8)	4.32 (1.13–16.47)	0.63 (0.5–0.8)
Impaired efficiency	90.9 (82.9–96)	76.9 (46.2–95)	96.4 (89.8–99.3)	55.6 (30.8–78.5)	3.94 (1.46–10.65)	0.12 (0.06–0.24)
Labial seal	80 (59.3–93.2)	79 (68.1–87.5)	55.6 (38.1–72.1)	92.3 (83–97.5)	3.8 (2.36–6.13)	0.25 (0.11–0.56)
Oral residue	78.4 (61.8–90.2)	75 (62.6–85)	64.4 (48.8–78.1)	85.7 (73.8–93.6)	3.14 (1.99–4.95)	0.29 (0.15–0.54)
Pharyngeal residue	74.7 (64–83.6)	66.7 (41–86.7)	91.2 (81.8–96.7)	36.4 (20.4–54.9)	2.24 (1.15–4.36)	0.38 (0.23–0.62)
Piecemeal swallow	91.1 (82.6–96.4)	77.3 (54.6–92.2)	93.5 (85.5–97.9)	70.8 (48.9–87.4)	4.01 (1.85–8.69)	0.11 (0.05–0.24)

Se, sensitivity; Sp, specificity; PPV, positive predictive value; NPV, negative predictive value; LHR, likelihood ratio; CI, confidence interval.

## Discussion

The main finding of this study was that the modified V-VST offers a high discriminating ability in detecting OD among neurological patients. We also found that OD was a highly prevalent condition in neurological patients and was characterized by a high prevalence of videofluoroscopic signs of impaired safety and efficiency of swallowing, including frequent silent aspirations. The modified V-VST showed 96.6% sensitivity and 83.3% specificity for OD. Consequently, the modified V-VST should be promoted as a validated screening tool for OD among neurological patients.

The accuracy of the V-VST was first validated against that of the VFSS by using a starch-based thickener (18). However, starch-based thickeners present some limitations in taste (25), viscosity stability, and solubility (28). In contrast with starch-based thickeners, xanthan gum thickeners have a better taste (38) and a stable viscosity over time (32) and are not affected by amylase (31). Specifically, starch-based thickeners, but not xanthan gum thickeners, imparted a considerable grainy texture and a starch flavor to all solvents. Xanthan gum thickeners imparted a significantly higher “slickness” than the starch-based thickeners. In addition, starch-based thickeners can hydrolyze upon contact with amylase in the saliva (26, 27) and break down during the oral preparatory and oral phases of deglutition, which may lead to the change in viscosity. Patients with OD present with increased oral transit duration and delayed swallowing response (7, 39, 40). Compared with the use of starch-based thickener, xanthan gum thickener creates new stable networks that maintain viscosity levels over time. Taken together, xanthan is a more effective choice for use as a beverage and food thickener for OD (31, 36).

In the V-VST, volumes were increased from 5 to 10 ml and 20 ml boluses in a progression of increasing difficulty. However, in previous clinical practice, we found that even with the smallest volume of 5 ml, many severe swallowing difficulty patients showed aspiration or penetration. Our study also provided evidence for this point. The risk of aspiration or penetration in patients leads to the development of a smaller initial volume. A 3 ml volume had been used in swallowing screening and assessment tests in previous studies. As early as 2003, Tohara et al. studied the accuracy of three non-videofluorographic tests for assessing the risk of aspiration, including the 3-ml water swallowing test (WST), in individuals with dysphagia (21). Since then, WST including a 3-ml volume has been observed in the next series of studies (22, 23). The modified WST (MWST), which used only 3 ml of water, yielded a sensitivity of 55.3% and a specificity of 80.8% for aspiration (23). Similarly, when performing bolus swallowing, boluses of 3, 5, 10, and 20 ml of different viscosities were used for the detection and characterization of multiple swallow behaviors in a recent study (24). These studies suggest that 3 ml is a common parameter in swallowing screening and assessment tests. Taken together, we added a 3 ml volume to the test as the initial volume.

In this study, we used VFSS to verify that unsafe swallowing was observed in 76.7, 62.2, 42.2, and 43.3% of patients during 5 ml swallows of water, mildly thick, moderately thick, and extremely thick viscosities, respectively. We also found that according to VFSS, the prevalence of safe swallowing varied significantly for the 3 ml and 5 ml swallows at mildly thick and moderately thick viscosities. For the modified V-VST, the prevalence of safe swallowing differed significantly for the 3 ml and 5 ml swallows of the water viscosity bolus, indicating that 3 ml swallows were enough to identify some patients with impaired safety, which could avoid aspiration and penetration of larger volumes in these patients. In addition, a 3-ml volume provides a potentially safe volume for patients who swallow unsafely in a 5 ml bolus and can better guide patients' oral feeding training.

We found that the prevalence of safe swallowing during 20 ml swallows was low (14.4–28.9%), which means that approximately 70% of the patients presented aspiration or penetration during 20 ml swallows. A noteworthy finding is that removing the results of the 20 ml swallows did not reduce the sensitivity of the test. Unsafe swallows can still be screened at a lower viscosity. Removing the 20 ml swallows can help avoid unnecessary aspiration or penetration.

Previous studies suggest that V-VST has good sensitivity (84.2–87.0%) and acceptable specificity (64.3–81.0%) in detecting impaired swallowing safety (17–19). A recent systematic and scoping review reported that V-VST has a 93.2% sensitivity and 81.4% specificity for detecting OD (20). Similarly, in our study, modified V-VST showed 85.2% sensitivity and 70% specificity for impaired safety, indicating a high discrimination ability. For aspiration, V-VST presented a sensitivity of 88.2–100% and a specificity of 28.0–71.4% (17–19). Consistently, our study revealed a sensitivity of 89.4% and a specificity of 70% to detect aspiration. Compared with aspiration, it is more difficult to detect penetration using those clinical signs. Guillén-Solà reported low sensitivity of 34.3% (17), while Clavé reported a sensitivity of 83.7% and specificity of 70.6 and 64.7%, respectively (18). Our study revealed a sensitivity of 66.7% and a specificity of 70% to detect penetration. These results showed that the modified V-VST presented high sensitivity and specificity to detect OD, impaired safety, and aspirations, clearly showing a high discrimination ability.

Due to the lack of standardized protocols and interpretation of findings, voice quality or oxygen desaturation showed limited sensitivity and specificity for detecting penetration/aspiration (41, 42). In our study, clinical signs of impaired safety of swallowing showed sensitivities of 65.4, 58.0, and 43.2% for cough, voice change, and desaturation by ≥3%, and specificities of 85%, 75%, and 90%, respectively. Cough, voice change, and oxygen desaturation are common clinical signs of impaired safety of swallowing (43, 44). In clinical practice, especially in V-VST, co-application of these signs can increase the sensitivity for detecting impaired safety (17). Consistently, in our study, the co-application of these signs increased the sensitivity and specificity for detecting impaired safety of the three signs, showing a sensitivity of 85.2% and specificity of 70% for unsafe swallowing. Taken together, at the bedside, the use of each of these measures in isolation is clearly not recommended but using them simultaneously is a helpful adjunct. These measures will provide more clinical data for speech-language pathologists to determine swallowing safety.

There are some limitations that should be taken into account when analyzing the results. In previous studies (18), researchers determined whether there was pharyngeal residue by oral reporting from patients. However, many patients have poor pharyngeal sensation and cannot report correctly, so the sensitivity and specificity of VVST to detect pharyngeal residue are only acceptable. The detection of pharyngeal residue in clinical screening should be modified. Future studies can use a suction tube to suck the epiglottic vallecula and pyriform sinus residues to judge whether there are residues in the pharynx. In conclusion, our study shows the high discriminating ability of the modified V-VST as a clinical bedside screening tool for OD among neurological patients.

## Data availability statement

The original contributions presented in the study are included in the article/supplementary material, further inquiries can be directed to the corresponding authors.

## Ethics statement

The studies involving human participants were reviewed and approved by the Ethics Committee of the Third Affiliated Hospital, Sun Yat-sen University. The patients/participants provided their written informed consent to participate in this study.

## Author contributions

HW and ZD designed the experiment. YL, GW, HW, JS, YZ, HC, XW, ZT, and MD involved in patients' evaluation and data collection. YL, GW, and HW wrote the manuscript. All authors read and approved the final manuscript.

## Funding

This study was supported by grants from the National Natural Science Foundation of China (No. 81972159).

## Conflict of interest

The authors declare that the research was conducted in the absence of any commercial or financial relationships that could be construed as a potential conflict of interest.

## Publisher's note

All claims expressed in this article are solely those of the authors and do not necessarily represent those of their affiliated organizations, or those of the publisher, the editors and the reviewers. Any product that may be evaluated in this article, or claim that may be made by its manufacturer, is not guaranteed or endorsed by the publisher.
